# Social threat, neural connectivity, and adolescent mental health: a population-based longitudinal study

**DOI:** 10.1017/S0033291725101384

**Published:** 2025-09-18

**Authors:** Dimitris I. Tsomokos, Henning Tiemeier, George M. Slavich, Divyangana Rakesh

**Affiliations:** 1 Department of Psychology & Human Development, UCL Institute of Education, University College London, London, UK; 2 Department of Neuroimaging, Institute of Psychology, Psychiatry & Neuroscience, King’s College London, London, UK; 3 Department of Social and Behavioral Sciences, Harvard T.H. Chan School of Public Health, Boston, MA, USA; 4 Department of Psychiatry and Biobehavioral Sciences, University of California, Los Angeles, CA, USA

**Keywords:** adolescent psychopathology, externalizing problems, functional connectivity, internalizing problems, neuroimaging, social safety theory

## Abstract

**Background:**

Although perceived threats in a child’s social environment, including in the family, school, and neighborhood, are known to increase risk for adolescent psychopathology, the underlying biological mechanisms remain unclear. To investigate, we examined whether perceived social threats were associated with the functional connectivity of large-scale cortical networks in early adolescence, and whether such connectivity differences mediated the development of subsequent mental health problems in youth.

**Methods:**

Structural equation models were used to analyze data from 8,690 youth (50% female, 45% non-White, age 9–10 years) drawn from the large-scale, nationwide Adolescent Brain Cognitive Development study that has 21 clinical and research sites across the United States. Data were collected from 2016 to 2018.

**Results:**

Consistent with Social Safety Theory, perceived social threats were prospectively associated with mental health problems both 6 months (standardized 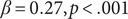 ) and 30 months ( 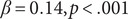 ) later. Perceived social threats predicted altered connectivity patterns within and between the default mode (DMN), dorsal attention (DAN), frontoparietal (FPN), and cingulo-opercular (CON) networks. In turn, hypoconnectivity within the DMN and FPN – and higher (i.e., less negative) connectivity between DMN-DAN, DMN-CON, and FPN-CON – mediated the association between perceived social threats and subsequent mental health problems.

**Conclusions:**

Perceiving social threats in various environments may alter neural connectivity and increase the risk of psychopathology in youth. Therefore, parenting, educational, and community-based interventions that bolster social safety may be helpful.

## Introduction

Adolescence is a uniquely important developmental period marked by significant neurobiological, psychological, and social changes. During this time, youth are particularly vulnerable to mental health problems that can have a lasting impact into adulthood (Sawyer et al., 2012; Solmi et al., 2022). In recent years, the rising prevalence of youth mental health problems has become a pressing public health concern (Jones et al., 2022; Racine et al., 2021). The Centers for Disease Control and Prevention reported that, in 2021, four out of 10 high school students in the United States struggled with persistent sadness or hopelessness, and more than one in six had made a suicide plan (CDC, 2023).

The factors that influence mental health are numerous, dynamic, and complex, ranging from the physical environment to social and cultural milieus, which interact with one another and with human biology (Schumann et al., 2024), consistent with bioecological (Bronfenbrenner, 2005) and biopsychosocial (Bolton, 2023) models of development and pathogenesis. Three fundamental social environments for children and adolescents – the family, school, and neighborhood – play a critical role in child and adolescent development (Barber & Olsen, 1997; Epstein & Sanders, 2002). Moreover, greater threat perception in these environments has been associated with heightened risk for youth psychopathology (Basu & Banerjee, 2020; Beyer et al., 2024; dos Santos, Santos, Machado, & Pinto, 2023; Huang, Edwards, & Laurel-Wilson, 2020; Rakesh, Allen, & Whittle, 2023; Raniti, Rakesh, Patton, & Sawyer, 2022; Repetti, Taylor, & Seeman, 2002; Tsomokos & Slavich, 2024; van Eldik et al., 2020).

Social Safety Theory (SST) provides a useful framework for understanding the roots of these mental health challenges (Slavich, 2020, 2022; Slavich, Roos, et al., 2023). In brief, SST posits that human behavior has evolved to detect and respond to environmental conditions that signal safety or threat. In contexts where individuals perceive their social environment as unsafe – due to conflict, violence, or instability – neurophysiological responses that confer survival benefits are triggered. Although these responses may be beneficial in the short term, chronic exposure to threats in the social environment (i.e. social threats) can prolong biological responses such as inflammation (Eisenberger et al., 2017; Slavich, Way, Eisenberger, & Taylor, 2010) and cause neurobiological changes that have long-term health effects (Allen et al., 2021; Chen & Nuñez, 2010; Morese et al., 2019; Slavich, Mengelkoch, & Cole, 2023; Uchino et al., 2018). Such physiological changes have, in turn, been related to changes in brain connectivity that have implications for mental health. For instance, inflammation has been associated with lower connectivity of corticostriatal circuits that regulate motivation and motor function and the ventromedial prefrontal cortex, implicated in emotion regulation (Alvarez, Hackman, Miller, & Muscatell, 2020; Goldsmith, Bekhbat, Mehta, & Felger, 2023; Miller, White, Chen, & Nusslock, 2021; Schrepf et al., 2018), which may increase individuals’ susceptibility to psychopathology (Rakesh, Dehestani, & Whittle, 2024).

Consistent with this work, emerging research suggests that functional connectivity in large-scale brain networks mediates the relation between social–environmental stressors and youth mental health outcomes (Andrews, Ahmed, & Blakemore, 2021; Berboth & Morawetz, 2021; Chahal, Gotlib, & Guyer, 2020; Holz et al., 2023; Jiang et al., 2021; Rakesh, Allen, et al., 2023; Rakesh, Kelly, et al., 2021). Resting-state functional Magnetic Resonance Imaging (rs-fMRI) is particularly well-suited for this analysis because it allows for the measurement of intrinsic functional connectivity patterns, which may mediate associations between perceived threats and affective states by reflecting stable neural alterations involved in emotional processing and self-regulation (McLaughlin, Sheridan, & Lambert, 2014; Rakesh et al., 2024). According to the Triple Network Model (Menon, 2011), for example, interactions among the Default Mode Network (DMN), Salience Network (SN), and Fronto-Parietal Network (FPN) play a role in self-referential processing, cognitive control and emotion regulation, and they have been implicated in a wide range of psychopathologies (Bertocci et al., 2023; Jones et al., 2023; Schumer et al., 2024; Thakuri, Bhattarai, Wong, & Chand, 2024).

It has been suggested that the SN detects the presence of salient stimuli and mediates the switch between the function of the DMN, which is responsible for internally oriented and self-referential thought, and the FPN (Seeley et al., 2007), which supports cognitive control and emotion regulation (Cole et al., 2013). Recent evidence also suggests that higher functional connectivity of the DMN and the Dorsal Attention Network (DAN) (Jirsaraie et al., 2024) is associated with internalizing and attention problems (Lees et al., 2021; Rakesh, Zalesky, & Whittle, 2023). Additionally, higher connectivity between the DMN and the Cingulo-Opercular Network (CON) – which, in some atlases, includes cortical regions such as the insula and anterior cingulate cortex, both crucial for emotion processing and regulation – has been linked to internalizing problems in preadolescents (Lees et al., 2021).

Social threats and a lack of perceived safety in the home, school, and neighborhood are potent stressors that may heighten emotional and physiological arousal (Slavich, O’Donovan, Epel, & Kemeny, 2010). Such stressors have been shown to activate neural circuits that process social and environmental cues, particularly those related to threat detection and emotion regulation (Chahal et al., 2022; Eisenberger & Cole, 2012; Rakesh et al., 2024; Sebastian, Viding, Williams, & Blakemore, 2010; Slavich & Cole, 2013). In the broader context of Adverse Childhood Experiences (ACEs) (McLaughlin et al., 2012), there is growing interest in understanding the mechanisms through which such experiences, particularly those related to threat, lead to harmful mental health outcomes (Kim & Royle, 2025; Schäfer et al., 2023). Yet, despite these advances, longitudinal studies that examine the neural mechanisms linking social threats to later psychopathology remain scarce (Whittle, Zhang, & Rakesh, 2025). Moreover, prior dimensional adversity research has largely examined threat within single domains, overlooking the influence of threat exposure across multiple environmental contexts (Schäfer et al., 2023). The current study addresses this critical gap by assessing concurrent social threat across family, school, and neighborhood domains, enabling a more nuanced and ecologically valid understanding of how social threats may shape adolescent development.

To address these issues, we explored two research questions. First, we investigated how perceptions of social threat (in the family, school, and/or neighborhood) during early adolescence were related to differences in functional connectivity within and between the DMN, DAN, FPN, CON, and SN. Second, we examined whether any such differences in connectivity mediate the influence of social threats on subsequent mental health problems. Using data from the Adolescent Brain Cognitive Development (ABCD) study – a large population-based developmental neuroimaging cohort from the United States (Casey et al., 2018; Saragosa-Harris et al., 2022) – we conducted an exploratory investigation in a socio-demographically diverse sample of 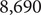 youth.

Based on the research summarized above, we hypothesized that perceived social threats would be associated with alterations in functional connectivity within and between the DMN, DAN, FPN, CON, and SN. Moreover, we hypothesized that these connectivity patterns would mediate the associations between social threats and mental health problems, including internalizing, externalizing, and attention difficulties 6 months later (as well as 30 months later). In additional analyses, we delineated the unique associations of social threats in the family, school, and neighborhood with brain and behavior outcomes. However, given the lack of prior studies delineating the effects of home, school, and neighborhood, we did not formulate specific hypotheses regarding the relative strength of the effects of these different life contexts on youths’ neural connectivity or mental health.

## Methods

The ABCD study recruited over 11,800 children aged 9–10 years (2016–2018) from a diverse sample across 21 sites, with 6-month follow-ups (Casey et al., 2018; Saragosa-Harris et al., 2022). By collecting comprehensive data – including neuroimaging, cognitive assessments and health evaluations – the study aims to characterize child brain and behavior development. Participants in our analysis were drawn from the baseline wave and 6-month and 30-month follow-up waves, from approximately age 10 through age 12.5 (see Table 1 for demographic characteristics). The study adheres to the Strengthening the Reporting of Observational Studies in Epidemiology (STROBE) guidelines for observational research (Von Elm et al., 2007).

**Table 1.** Demographic profile of the analytic sample at baseline

Characteristics	Sample (*N* = 8,690)
Exact age (months), mean (SD)	119 (8)
Sex, *n* (%)	
Female	4,318 (50)
Male	4,372 (50)
Race/ethnicity, *n* (%)	
Asian	175 (2.0)
Black	1,111 (13)
Hispanic	1,706 (20)
Other	900 (10)
White non-Hispanic	4,796 (55)
(Missing)	2
Area deprivation, mean (SD)	39 (26)
(Missing)	588
Parental age (years), mean (SD)	40 (7)
(Missing)	55
Parental education, *n* (%)	
1 (higher education degree)	4,892 (56)
0 (no degree)	3,788 (44)
(Missing)	10
Parental mental health, mean (SD)	21 (18)
(Missing)	2
Unsafe neighborhood, *n* (%)	
0 (No)	7,887 (91)
1 (Yes)	790 (9.1)
(Missing)	13
Unsafe school, *n* (%)	
0 (No)	8,164 (94)
1 (Yes)	513 (5.9)
(Missing)	13
Family conflict, mean (SD)	2 (2)
(Missing)	12
Social threats (total score), mean (SD)	0.37 (0.49)
(Missing)	14

**Table 2.** Main results of the 15 models ( 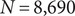 , adjusted, imputed) for the resting-state functional connectivity outcomes (within and between the five focal networks) at age 10.5 (first follow-up wave) regressed on total perceived social threat at age 10 (baseline)

Brain connectivity outcome	Coefficient^a^	Standardized^b^	*p*-value^c^	FDR-adjusted *p*-value^c^
Within DMN	−0.004	−0.035	0.001	**0.003**
Within DAN	−0.004	−0.028	0.012	**0.025**
Within FPN	−0.005	−0.043	<0.001	**<0.001**
Within CON	−0.006	−0.045	<0.001	**<0.001**
Within SN	0.006	0.024	0.036	0.062
DMN-DAN	0.005	0.050	<0.001	**<0.001**
DMN-FPN	−0.000	−0.004	0.737	0.736
DMN-CON	0.003	0.030	0.005	**0.011**
DMN-SN	−0.002	−0.020	0.064	0.087
DAN-FPN	0.001	0.016	0.138	0.172
DAN-CON	−0.002	−0.022	0.041	0.062
DAN-SN	0.001	0.012	0.290	0.323
FPN-CON	0.003	0.035	0.001	**0.003**
FPN-SN	−0.001	−0.011	0.302	0.323
CON-SN	0.003	0.023	0.039	0.061

*Note:* Model fit indices have been omitted since these models are fully saturated. Results for all the covariates (sex, area deprivation, parental education, parental mental health, fMRI scanner type and motion during scans) are included in Section A of the SOM.

a Unstandardized coefficients.

b Standardized coefficients.

c *p*-values before and after controlling for the FDR (bold values indicate significance even after applying the correction).

### Perceived social threat (age 10)

We conceptualized perceived social threat as threats experienced in the child’s various social environments (i.e. home, school, and neighborhood). The exposure – total perceived social threats at baseline – was a numerical variable ranging from 0 to 3, with 3 representing the most negative perceptions of social threats from all environments, which we obtained by summing three separate measures: (a) *family conflict* scale (Moos & Moos, 1994) (normalized from 0 to 1, with higher values corresponding to more conflict); (b) *unsafe school* (dichotomized after reverse-coding), a self-report item (“I feel safe at my school”) from the School Risk and Protective Factors section (Arthur et al., 2007); and (c) *unsafe neighborhood* (dichotomized, reverse-coded), a self-report item (“My neighborhood is safe from crime”) from Neighborhood Safety/Crime (Mujahid, Diez Roux, Morenoff, & Raghunathan, 2007). More details about all the variables can be found in the Supplemental Online Material (SOM, 2025).

### Functional connectivity (age 10)

Imaging procedures were thoroughly detailed in Casey et al. (2018). Participants underwent scanning at multiple sites following standardized protocols, completing four or five 5-minute resting-state scans (eyes open) to obtain at least 8 minutes of low-motion data. Further details are available in the Supplementary Information (SI) and Hagler et al. (2019). Preprocessing was conducted by the ABCD Data Analysis and Informatics Core using the standardized ABCD pipeline – see Hagler et al. (2019) for details. Subsequently, fMRI time series were mapped onto FreeSurfer’s cortical surface. Connectivity within and between networks was then computed using Pearson correlation, based on the Gordon parcellation scheme (Gordon et al., 2016) across 12 predefined resting-state networks. In our study, we analyzed connectivity within and between the Cingulo-Opercular Network (CON), Dorsal Attention Network (DAN), Default Mode Network (DMN), Frontoparietal Network (FPN), and Salience Network (SN), which results in 15 connectivity variables of interest. Connectivity values were Fisher Z-transformed.

### Mental health problems at age 10.5 (and 12.5)

The primary outcome was the total score from the internalizing, externalizing, and attention problem subscales (i.e. total mental health symptoms) from the youth self-report Brief Problem Monitor (Achenbach, 2009), drawn from the first follow-up wave (6 months after baseline), and in secondary analyses, we also drew the same measure from the 30-month follow-up. Internalizing problems consisted of six items on a scale from 0 (*not true*) to 2 (*very true*), with a higher total score indicating more problems. Externalizing problems consisted of seven items on the same scale, and attention problems consisted of six items. Therefore, the mental health problems measure consisted of 19 items, with a total score from 0 to 38.

### Covariates at age 10 years or earlier

A variety of factors were included as confounders based on their known association with both the exposures and outcomes studied here while keeping the model as parsimonious as possible (Saragosa-Harris et al., 2022; SOM, 2025; Whittle et al., 2025). Biological *sex* was male or female. Area deprivation index (ADI) was a derived composite variable reflecting neighborhood disadvantage (national percentile score) (Kind et al., 2014). Neighborhood disadvantage has been associated with perceived social threats (Arthur et al., 2007; Chahal et al., 2022; Eisenberger & Cole, 2012; Holz et al., 2023; Huang et al., 2020; Rakesh et al., 2024), brain structure and function (Rakesh & Whittle, 2021; Rakesh, Whittle, Sheridan, & McLaughlin, 2023; Rakesh, Zalesky, & Whittle, 2021, 2022), and youth psychopathology (Beyer et al., 2024; Epstein & Sanders, 2002; Mujahid et al., 2007; Repetti et al., 2002; Schumann et al., 2024; Whittle et al., 2025). *Parental education* was a derived, dichotomous variable based on the (responding) parent’s highest educational attainment at baseline (tertiary education or not), which has been associated with both brain function and mental health of offspring (Jiang et al., 2021; Rakesh et al., 2024; Rakesh & Whittle, 2021). *Parental mental health* was the (responding) parent’s total score on the self-reported Total Problems ASR-ASEBA for broad psychopathology (0–154) (Achenbach, 2009), associated with youth brain development and mental health (Holz et al., 2023; Jirsaraie et al., 2024; Rakesh et al., 2024). We also accounted for the *scanner model used*, and *fMRI motion* measured average framewise displacement (continuous variable in mm) during the scan. *Race/ethnicity* was dichotomized as non-Hispanic White versus non-White (based on White, Black, Hispanic, Asian, and Other). Finally, children’s total mental health symptoms at baseline were obtained from the parent-reported Child Behavior Checklist with 113 items (we used the normalized T-score, a continuous variable) (Achenbach, 2009) as youth-reported mental health was not assessed at baseline.

### Analytic sample

Of the 11,868 participants at baseline, we excluded 681 participants with incomplete neuroimaging data and a further 1,839 based on recommended exclusion criteria for resting-state data (9,348 participants remaining). We excluded 656 participants with incomplete data on total mental health symptoms at the 6-month follow-up, and two participants who were intersex at birth. Therefore, the final main analytic sample consisted of 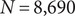 participants (SI Figure S1). For the additional analysis in which mental health outcomes were measured at the 30-month follow-up, we had 9,348 participants with valid neuroimaging data and excluded 1,773 with incomplete outcome measures (and two intersex participants), so that the final analytic sample in this case was 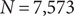 . Table 1 summarizes key demographic characteristics of the analytic sample at baseline.

### Main analysis

**Figure 1.**
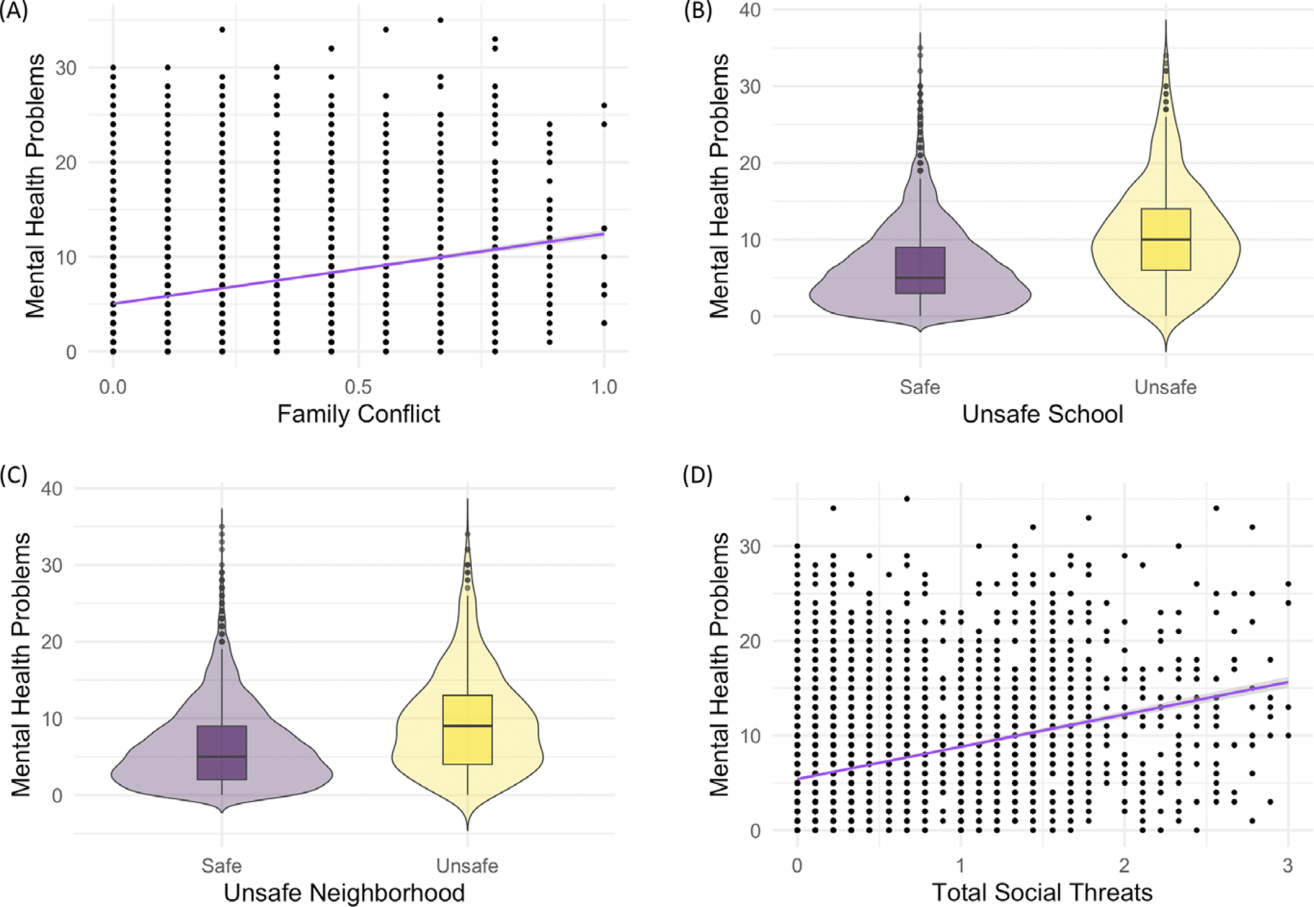
Relations between perceived social threats at baseline (age 10) and total mental health symptom scores 6 months later ( 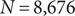 ). (a) Scatterplot for family conflict and mental health; (b) violin boxplot for safe/unsafe school environment and mental health; (c) violin boxplot for safe/unsafe neighborhood and mental health; (d) scatterplot between overall perceived social threats from family conflict, school, or neighborhood, and subsequent mental health symptom scores. Family conflict ranges from 0 to 1 (0 = no conflict), whereas school and neighborhood unsafety are dichotomous (0 = safe, 1 = unsafe).

In a preliminary analysis, we examined the sample’s demographic characteristics (Table 1 and SI, Table S1), variable descriptives (SI), and correlations between the continuous variables (SI, Table S2). We performed crude bivariate analyses to gain insights into the relationship between perceived social threats and mental health scores (Figure 1). In this initial step, we also analyzed patterns of missingness in the data, which, in turn, determined our approach to data imputation. Second, multiple regression models explored the association between perceived social threats and functional connectivity, adjusting for confounders (there was a separate model for each rs-fMRI connectivity variable). Perceived social threats were a manifest variable (the sum of three scores from observable variables), not a latent one. For those cases where perceived social threats were associated with connectivity, structural equation models were used to test whether brain connectivity mediated the relation between perceived social threats at baseline and total mental health symptoms 6 months later. Although our predictor variable was assessed at the first survey wave, as was neuroimaging, participants’ perceptions of safety were captured via questionnaires and indicate a general sense of safety over a recent period (which precedes the scans). This establishes a minimal temporal sequence, in which perceived threats preceded the measurement of neural connectivity.

The preliminary analyses showed that the data were not Missing Completely at Random (MCAR); therefore, data imputation was warranted to avoid non-response and attrition bias (as a complete case analysis is only warranted when the mechanism is MCAR) (Hayes & Enders, 2023). Missing values were imputed using Full Information Maximum Likelihood, and results were calculated both before and after applying a false discovery rate (FDR) correction using the procedure of Benjamini and Hochberg. An FDR correction was applied in as many models as were fitted at each step – that is, in the initial step that involved five focal networks, the correction was applied on 15 variables (all combinations of the five networks), and similarly for all the mediation models as well. For all calculations, we used R 4.4.1 (R Core Team, 2021) and the *lavaan* package (Rosseel, 2012) with Maximum Likelihood Estimator for robust standard errors and confidence intervals. To assess model fit, we use the standard metrics, cutoffs, and recommendations in Hu and Bentler (1999), and the full model fit details in every case are reported in the supplement.

### Sensitivity and additional analyses

The parsimonious models of the main analysis were refitted with two additional covariates: child race/ethnicity, which is related to various forms of discrimination and adverse social experiences (Jorgensen et al., 2023; Umberson et al., 2014), and child mental health problems at baseline (parent-reported). We added these here – as opposed to including them in the main model – because, first, the child’s mental health problems were reported by the adult respondent (not the child, who self-reported the outcomes), and second, because there is evidence of a robust association between race/ethnicity and adversity (Harnett et al., 2024), aspects of which are already controlled for in our core model through area deprivation and parental education. We also allowed for random effects after nesting participants within families and within 21 research sites. Finally, two additional (secondary outcomes) analyses were performed, investigating the specificity of perceived social threats and mental health problems: first, the outcome was delineated into internalizing, externalizing, and attention problems; second, perceived social threats were delineated into threats arising from family conflict, school unsafety, and neighborhood unsafety, controlling for each other in the same model (SI for details).

## Results

Table 1 summarizes key demographic characteristics of the participants in the baseline wave, approximately aged 10 years (50% female, 45% non-White or Hispanic participants).

### Social threats and subsequent mental health

First, we examined bivariate associations between perceived threat due to (A) family conflict, (B) unsafe school, (C) unsafe neighborhood, or (D) perceived social threats from all three contexts, and subsequent mental health (Figure 1). Note that total social threats (i.e. case D) is the main independent variable, operationalized as the sum of the normalized family conflict scale (a numerical variable ranging from 0 to 1), and the dichotomized measures for unsafe school and unsafe neighborhood environments. In each of these cases (A–D), perceived social threats were positively associated with subsequent mental health problems. A sample bias analysis and correlations between numerical variables are provided in Tables S1 and S2 (SI).

As hypothesized, these direct associations were robust even in the full regression model (*N* = 8,690 with imputation), where we adjusted for confounders (i.e. biological sex, area deprivation, parental education, and parental mental health), and controlled for the FDR across models (standardized 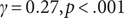 for total social threats, i.e. for case A). Importantly, this association remained significant in models where total social threats predicted mental health problems 2.5 years later, even after controlling for confounders and the FDR ( 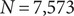 ), with standardized 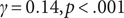 . In additional sensitivity analyses described in the Supplementary Information and SOM (Sections C, D), we confirmed that adjusting for child mental health problems at baseline (parent-reported) and race/ethnicity did not alter these findings; using exact child age – and nesting children within families and the study’s sites – also did not impact the findings. For all analyses, we confirmed key assumptions with regard to sufficient statistical power, multivariate normality, linearity, absence of collinearity among confounders, and independence of error terms, as explained in the Supplementary Information (SI text and Figure S3).

### Perceived social threats and brain connectivity

Second, we tested associations between total perceived social threats and 15 within and between network connectivity variables at baseline (*N* = 8,690), adjusting for biological sex, area deprivation, parental education, parental mental health, scanner model, and framewise displacement, and controlled for the FDR. Higher levels of perceived social threat were associated with lower connectivity within the DMN, DAN, FPN, and CON, and higher connectivity (i.e., less negative connectivity) between DMN-DAN, DMN-CON, and FPN-CON (Table 2; and Section A of the SOM). Moreover, sensitivity analyses showed that these findings were robust to additionally adjusting for the child’s baseline mental health problems and race/ethnicity, as well as to exact age and clustering within families and imaging sites (SOM, Sections C and D). In further exploratory analyses, we also examined whether sex or race/ethnicity moderated the associations but found no significant interaction effects (SOM, Appendix 6).

### The role of brain connectivity in the association between perceived social threats and subsequent mental health problems

**Figure 2.**
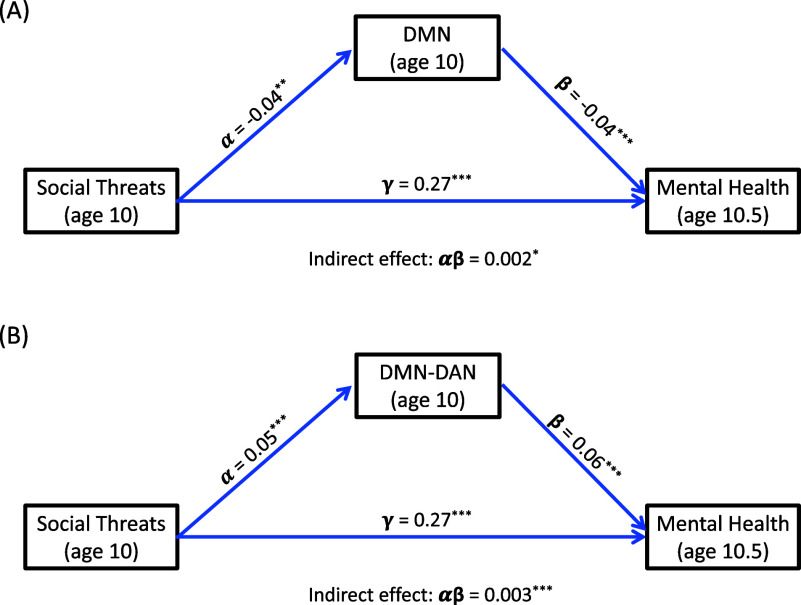
Simplified diagram of the relations between perceived social threats at baseline (age 10 years), brain connectivity, and total mental health symptom scores 6 months later ( 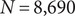 with data imputation). (a) Perceived social threats at age 10 degrade subsequent mental health, and this association is partially mediated by the within-network connectivity of the Default Mode Network (DMN). (b) Social threats degrade adolescent mental health, and this association is partially mediated by the between-network connectivity of the DMN and Doral Attention Network (DAN). Standardized path coefficients are shown (for models adjusted for sex, area deprivation, parental education and mental health at baseline, fMRI machine type and motion during scans, after controlling the FDR).

The seven connectivity variables (DMN, DAN, FPN, CON, DMN-DAN, DMN-CON, and FPN-CON) that were found to be significantly associated with perceived social threats were then tested as mediators in the prospective association between perceived social threats and total mental health problems 6 months later (see Figure 2; Table 3; SI Tables S3 and S4; and Section B of the SOM). We found significant indirect effects for lower connectivity within the DMN and FPN areas, and higher connectivity (i.e., less negative connectivity) between DMN-DAN, DMN-CON, and FPN-CON, even after controlling for all confounders and applying the FDR. As above, adding the child’s baseline mental health problems and race/ethnicity as covariates in these mediation models (in both the *a*-paths and *b*-paths) did not alter these findings (SI, Table S7; the only substantial difference in this case occurred for the mediation effect through DMN, for which we obtained a marginal FDR-adjusted *p*-value).

**Table 3.** Results for the structural equation models testing whether each of the seven connectivity variables that remained significant after FDR corrections in the initial analysis (Table 1) mediate the association between perceived social threats at baseline and total mental health problems 6 months later ( 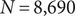 , adjusted, imputed)

Brain network	Social threats ➔ Mental health (*c*-path)	Social threats ➔ Connectivity (*a*-path)	Connectivity ➔ Mental health (*b*-path)	*ab* (indirect effect)
	Coeff^a^ (CI)^b^	Coeff^a^ (CI)^b^	Coeff^a^ (CI)^b^	Coeff^a^ (CI)^b^
Within DMN	2.90^***^ (2.62 to 3.18)	−0.00^***^ (−0.01 to −0.00)	−3.95^***^ (−5.83 to −2.07)	0.02* (0.00 to 0.03)
Within DAN	2.90^***^ (2.62 to 3.18)	−0.00* (−0.01 to −0.00)	−2.15^**^ (−3.72 to −0.59)	0.01 (−0.00 to 0.02)
Within FPN	2.90^***^ (2.62 to 3.18)	−0.00^***^(−0.01 to −0.00)	−3.37^***^ (−5.22 to −1.52)	0.02^**^ (0.00 to 0.03)
Within CON	2.90^***^ (2.62 to 3.18)	−0.01^***^(−0.01 to −0.00)	−1.55* (−3.07 to −0.04)	0.01 (−0.00 to 0.02)
DMN-DAN	2.88^***^ (2.60 to 3.16)	0.01^***^ (0.00 to 0.01)	5.42^***^ (3.44 to 7.40)	0.03^***^ (0.01 to 0.05)
DMN-CON	2.89^***^ (2.61 to 3.17)	0.00^**^ (0.00 to 0.01)	5.62^***^ (3.65 to 7.60)	0.02* (0.00 to 0.03)
FPN-CON	2.90^***^ (2.62 to 3.18)	0.00^**^ (0.00 to 0.01)	4.04^**^ (1.75 to 6.32)	0.01* (0.00 to 0.02)

a Unstandardized coefficients (^***^p < 0.001; ^**^p < 0.01; *p < 0.05); indirect effects in bold remained significant after controlling for the FDR across all seven models.

b 95% confidence intervals. Tables S3 and S4 (SI document) include the coefficients for all covariates and the model fit indices (all fit indices were excellent).

In a secondary analysis, we disaggregated mental health problems into internalizing, externalizing, and attention problems (Appendices 1–3 of the SOM). Lower connectivity within the DMN and lower negative connectivity between DMN-DAN and DMN-CON mediated the association between social threats and internalizing symptoms. Crucially, however, for externalizing problems, there were no significant indirect effects. In the case of attention problems, there were significant indirect effects for the connectivity of DMN, FPN, CON, DMN-DAN, DMN-CON, and FPN-CON (see SI Tables S5 and S6).

### Secondary analysis on specificity of perceived social threats

**Figure 3.**
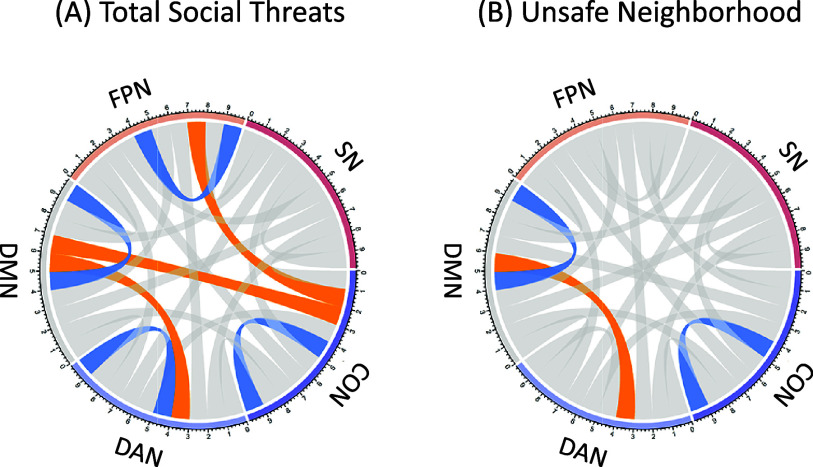
Chord diagram of brain connectivity as a function of social threats at age 10, comparing the cases of total social threats and neighborhood threats ( 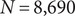 after imputation, for adjusted models). (a) Total social threats predict lower connectivity within the default mode (DMN), dorsal attention (DAN), cingulo-opercular (CON), frontoparietal (FPN) networks, and higher (i.e., less negative) connectivity between DMN-DAN, DMN-CON, and FPN-CON. (b) Perceived social threats arising from the neighborhood predict lower connectivity within the DMN and CON, and higher (i.e., less negative) connectivity between DMN-DAN, whereas social threats arising from the family or school are not associated with altered functional connectivity.

We further conducted an analysis whereby we tested associations among perceived family conflict, unsafe school, and unsafe neighborhood environments (controlling for each other in the same model), functional connectivity, and mental health (Table 4; Appendices 4 and 5 of the SOM). The results are summarized visually in Figure 3. Perceived neighborhood unsafety was associated with lower connectivity within the DMN and higher connectivity (i.e., lower negative connectivity) between the DMN-DAN, which mediated the association between perceived neighborhood unsafety and subsequent mental health symptoms. Notably, for the direct effects in these models – that is, associations between each source of perceived social threats and mental health scores – family conflict was most strongly related to mental health ( 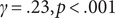 ), followed by school unsafety ( 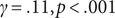 ), and then followed by neighborhood unsafety ( 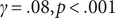 ). A formal statistical comparison between these coefficients revealed that the path from family conflict to mental health problems was indeed statistically stronger than the other two paths (see Supplementary Information).

**Table 4.** Results for two key models testing whether functional connectivity within the DMN and between DMN-DAN mediates the link between perceived social threats from (i) family, (ii) school, and (iii) neighborhood at baseline, and mental health problems 6 months later ( 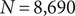 , models adjusted with covariates and imputed for missing data)

	Within DMN	DMN-DAN
	Coeff^a^ (CI)^b^	Coeff^a^ (CI)^b^
Total mental health problems		
Family conflict *(c_1_)*	5.83^***^ (5.28 to 6.37)	5.80^***^ (5.25 to 6.34)
Unsafe school *(c_2_)*	2.46^***^ (1.88 to 3.03)	2.45^***^ (1.87 to 3.02)
Unsafe neighborhood *(c_3_)*	1.42^***^ (0.96 to 1.88)	1.41^***^ (0.95 to 1.86)
Sex: Female	−0.69^***^ (−0.90 to −0.48)	−0.70^***^ (−0.91 to −0.49)
Area deprivation	0.01^***^ (0.01 to 0.02)	0.01^***^ (0.01 to 0.02)
Parental education	−0.64^***^ (−0.87 to −0.40)	−0.63^***^ (−0.86 to −0.40)
Parental mental health	0.04^***^ (0.03 to 0.04)	0.04^***^ (0.03 to 0.04)
Within DMN (*b*)	−4.16^***^ (−6.02 to −2.30)	
DMN-DAN (*b*)		5.45^***^ (3.48 to 7.42)
Functional connectivity		
Family conflict *(a_1_)*	−0.00 (−0.00 to 0.01)	0.01* (0.00 to 0.01)
Unsafe school *(a_2_)*	−0.00 (−0.01 to 0.00)	0.00 (−0.00 to 0.01)
Unsafe neighborhood *(a_3_)*	−0.01^**^ (−0.01 to −0.00)	0.01^**^ (0.00 to 0.01)
Sex: Female	0.02^***^ (0.01 to 0.02)	−0.01^***^ (−0.01 to −0.01)
Area deprivation	−0.00^***^ (−0.00 to −0.00)	0.00^***^ (0.00 to 0.00)
Parental education	0.00 (−0.00 to 0.00)	−0.00 (−0.00 to 0.00)
Parental mental health	−0.00 (−0.00 to 0.00)	0.00* (0.00 to 0.00)
	**Indirect effects** Coeff^a^ (CI)^b^	
*a_1_b* (family)	0.00 (−0.02 to 0.02)	0.03* (0.00 to 0.06)
*a_2_b* (school)	0.02 (−0.00 to 0.04)	0.02 (−0.00 to 0.05)
*a_3_b* (neighborhood)	0.03* (0.01 to 0.05)	0.03^**^ (0.01 to 0.06)
	**Fit indices** ^c^	
Robust CFI	0.99	0.99
Robust TLI	0.97	0.97
Robust RMSEA	0.02	0.02
SRMR	0.00	0.00

a Coeff = Unstandardized coefficients (^***^p < 0.001; ^**^p < 0.01; *p < 0.05; bold indicates that key coefficients remain significant after controlling for the FDR in three models with 10 paths each). Results for the neuroimaging covariates (fMRI scanner and motion during scans) are included in the SOM, Appendices 4 and 5).

b CI: Confidence interval (95% confidence level).

c Abbreviations: CFI: comparative fit index; TLI: Tucker–Lewis index; RMSEA: root mean square error of approximation; SRMR: standardized root mean square residual.

### Secondary analysis using a longer timeframe

In this additional analysis, we explored a longer timeframe, so that T1 (social threats and neuroimaging, age 10) ➔ Time 2 (mental health, age 12.5). As hypothesized, the association between perceived social threats and mental health symptoms 2.5 years later was mediated by greater connectivity (i.e., less negative connectivity) between the DMN-DAN. When social threats were delineated based on context and considered in the same model, DMN-DAN connectivity mediated the association for social threats arising from the neighborhood, but not the family or school environments.

## Discussion

These findings provide compelling new evidence that the perception of social threats in early adolescence is associated with differences in functional connectivity within and between large-scale cortical networks, such as the default mode and dorsal attention networks, and that these differences are, in turn, associated with the development of subsequent mental health problems. More specifically, perceived social threats at the cusp of adolescence (age 10) significantly predicted self-reported total mental health problems 6 months later (and 30 months later, at age 12.5) with small-to-moderate effect sizes. Further, perceived social threats were significantly associated with lower connectivity within the DMN and FPN, and higher (i.e., less negative) connectivity between DMN-DAN, DMN-CON, and FPN-CON, and these altered connectivity patterns, in turn, mediated the effect of perceived social threats on subsequent internalizing problems and attention problems but not externalizing problems.

When we delineated perceived social threats into those arising from the family, school, or neighborhood environments, and all three were considered simultaneously in the same model (controlling for one another), threats arising from the family were the strongest predictor of subsequent mental health problems, followed by school and then neighborhood. However, in terms of mediation effects through altered patterns of neural connectivity, only the neighborhood environment was robustly associated with DMN and DAN connectivity (and functional connectivity within the DMN and between DMN-DAN mediated the association between perceived neighborhood unsafety and later mental health problems).

Taken together, these results provide evidence that perceived social threats due to experiences of family conflict in the home environment, or unsafe schools and neighborhoods, can instil negative social safety schemas (Slavich, 2020; Slavich, Way, et al., 2010) in early adolescence, which is a period typified by dynamic brain development (Rakesh et al., 2024) and the onset of mental health disorders (Sawyer et al., 2012). Social safety schemas, in turn, contribute to the persistence of mental health problems across adolescence and, therefore, the present work supports existing evidence that such maladaptive social-cognitive schemas are a valid target for clinical intervention (Alley, Tsomokos, Mengelkoch, & Slavich, 2025). Targeting such schemas is especially important in this age group, where environmental factors – such as victimization – are both influential and potentially modifiable, particularly for the substantial proportion of youth (approximately one in three) exposed to more severe forms of victimization (Fisher et al., 2015).

The associations uncovered between social threats and connectivity within and between the five focal networks are in line with the Triple Network Model (Menon, 2011), which implicates these brain networks in social-cognitive and emotional dysregulation. Our results suggest a potential neural mechanism by which social threats heighten sensitivity to environmental stressors and impair regulatory processes, as expected by Social Safety Theory (Slavich, 2020). Crucially, given that resting-state functional connectivity increases during normative development (Khundrakpam et al., 2016; Rakesh et al., 2024; Truelove-Hill et al., 2020), lower connectivity within these networks, including the DMN, may suggest that perceived threats in youths’ social environments are associated with measurable deviations from typical neurodevelopment in early adolescence (Rakesh, Kelly, et al., 2021). Our findings, thus, provide novel insights into the specificity of neural substrates of risk factors in the social environment and the consequences of maladaptive social safety schemas on adolescent mental health.

These findings are consistent with prior studies that have elucidated associations between childhood maltreatment and alterations in resting-state functional connectivity. In a systematic review, Gerin et al. (2023) reported evidence linking childhood maltreatment with heightened connectivity of the amygdala with key nodes in the salience, default mode, and prefrontal regulatory networks. Crucially, these patterns of altered connectivity were associated with poor cognitive and social functioning and predicted future psychopathology. Even though we focused on the Triple Network Model and did not investigate connectivity with subcortical brain areas, our results similarly highlight alterations within and between networks critical for emotion regulation and social-cognitive function, such as the DMN, FPN, and DAN. This points to a potential commonality in neural mechanisms through which various forms of social threat and maltreatment may influence developmental psychopathology. Our findings are also consistent with recent evidence of lower within-network DMN connectivity for children experiencing their neighborhoods as threatening (Vargas, Rakesh, & McLaughlin, 2025).

Additionally, longitudinal studies show that within-network connectivity of the DMN, which supports self-referential cognitive processing and is widely implicated in autobiographical memory, prospection, and theory of mind (Spreng, Mar, & Kim, 2009), increases during childhood and adolescence (Fan et al., 2021; Rakesh, Sadikova, & McLaughlin, 2025). Despite the fact that high within-network connectivity of the DMN has been associated with affective disorders in adults (Sambataro, Wolf, Pennuto, Vasic, & Wolf, 2014; Whitfield-Gabrieli & Ford, 2012; J. Zhang et al., 2023), often signaling increased rumination and other internalizing symptoms, decreased within-DMN connectivity has been linked to emotion dysregulation (Ernst et al., 2019), conduct problems (Zhou et al., 2016), attention problems (Broulidakis et al., 2022; Fateh et al., 2023), and shorter sleep duration is associated with later behavioral problems in this age group (Zhang, Geier, House, & Oshri, 2025). Therefore, the results of the present study – for instance, that the DMN’s lower within-network connectivity was found to mediate the association between perceived social threats and subsequent internalizing and attention problems – are in line with several findings recently reported in the literature.

### Strengths, limitations, and future directions

This study has several strengths, including a large longitudinal sample, rigorous adjustment for confounders, and theoretically guided analyses. However, a few limitations should also be noted. First, the observational design of the study precludes causal inferences. Although we adjusted for several potential confounders, unmeasured variables may still influence the associations observed. Crucially, it is not possible to identify the causal direction of the observed association between perceived social threats and differences in functional connectivity. Second, although we controlled for an objective measure of area deprivation (and additionally for race and ethnicity) and gained further insights through moderation analyses involving race and ethnicity, the interplay between low socioeconomic status, race/ethnicity, and various other stressors experienced by the family has not been captured here. This is a complex area that deserves further attention, especially given recent findings on the close associations between race/ethnicity and adversity (Harnett et al., 2024), and the problems associated with the uncritical use of race/ethnicity as a confounder in all models (Cardenas-Iniguez & Gonzalez, 2024; Harnett & Dumornay, 2024). Third, our study only measured perceived social threats. Objective measures of threat, such as contact with child protective services, official records of bullying and victimization, and neighborhood crime rates, may yield differing results. Future work should compare subjective versus objective measures of social threats.

Four, although the ABCD Study provides a diverse sample, the generalizability to other environments and cultural contexts may be limited. Replicating the study in different countries and settings would strengthen the external validity of the present findings. Fourth, key measures were self-reported; that is, both measures of perceived social threats (exposure) and total mental health problems (outcome) were youth-reported. Although there is obvious simplicity and other benefits to such self-reports (Corneille & Gawronski, 2024), it would also be beneficial to have independent measures of social safety and objective clinical outcomes, such as linked hospital records or clinician-rated diagnoses.

Finally, the clinical relevance of our findings must be discussed in light of the effect sizes reported in this study. In general, the associations between social safety perceptions and subsequent mental health problems in youth (both 6 months later and 2.5 years later) were relatively small. Practically, a 1-point increase in perceived social threats (on a scale from 0 to 3) was associated with a 3-point increase in the total mental health problems score (on a scale from 0 to 38). However, despite the moderate effect size (standardized 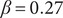 ), perceived social threats can lead to the development of maladaptive social safety schemas and contribute to the persistence of mental health problems during adolescence (Alley et al., 2025). Such social safety schemas are, therefore, a valid target for clinical interventions, given that their effect can be compounded over time (clinical implications of our work are discussed below). In addition, in the context of adolescent mental health, a small effect may, in fact, be meaningful and impactful from the perspective of public health, as explained in Funder and Ozer (2019) and Carey, Ridler, Ford, and Stringaris (2023).

Future research on this topic should examine the role of the neighborhood environment further. As a result of the present work, one hypothesis would be that youths’ perceived neighborhood unsafety is more robustly associated with the connectivity patterns of the DMN and DAN because its nature is inherently less predictable and broader compared to the home or school environments, giving rise to different types of social safety stressors. Our findings also suggest that the physiological pathways that mediate the associations between family and school environments and subsequent youth mental health problems may involve other biological mechanisms (beyond the neural mechanisms investigated here), such as epigenetic changes and chronic inflammation (Furman et al., 2019).

### Implications for public health

Notwithstanding these limitations, the present findings have implications for public health interventions aimed at improving youth mental health. Crucially, the results highlight the significance of perceived social threats across contexts in shaping subsequent mental health and begin to elucidate neurocognitive mechanisms underlying these links. Youth perceptions of social threats coming from the family environment, school setting, and the neighborhood were related to subsequent mental health problems and to the functional connectivity of key brain networks that mediate these effects. Therefore, public health efforts to improve these sources of social threat, especially in late childhood and early adolescence, could also be informed by and aligned with brain health research. Consistent with this expectation, family-level interventions that support positive interpersonal relationships – for instance, through parenting programs – have shown positive changes in youth’s brain structure and behavior (O’Brien et al., 2023; Whittle et al., 2014), and our findings suggest that functional brain connectivity may also reflect the impact of such interventions.

## Supporting information

Tsomokos et al. supplementary material Tsomokos et al. supplementary material

## Data Availability

Data used in the preparation of this article were obtained from the Adolescent Brain Cognitive Development^SM^ (ABCD) Study (https://abcdstudy.org), held in the NIMH Data Archive (NDA). This is a multisite, longitudinal study designed to recruit more than 10,000 children aged 9–10 and follow them over 10 years into early adulthood. The ABCD Study^®^ is supported by the National Institutes of Health and additional federal partners under award numbers U01DA041048, U01DA050989, U01DA051016, U01DA041022, U01DA051018, U01DA051037, U01DA050987, U01DA041174, U01DA041106, U01DA041117, U01DA041028, U01DA041134, U01DA050988, U01DA051039, U01DA041156, U01DA041025, U01DA041120, U01DA051038, U01DA041148, U01DA041093, U01DA041089, U24DA041123, U24DA041147. A full list of supporters is available at https://abcdstudy.org/federal-partners.html. A listing of participating sites and a complete listing of the study investigators can be found at https://abcdstudy.org/consortium_members/. ABCD consortium investigators designed and implemented the study and/or provided data but did not necessarily participate in the analysis or writing of this report. This manuscript reflects the views of the authors and may not reflect the opinions or views of the NIH or ABCD consortium investigators.

## Supplementary material

The supplementary material for this article can be found at http://doi.org/10.1017/S0033291725101384.

## Code availability

Details of all variable names, analytic steps, and the complete output of our R code are publicly available (SOM, 2025) on the Open Science Framework website: https://osf.io/7xewv/ (D.I.T. accessed the data and wrote the code; D.R. accessed the data and wrote some parts of the code).
