# Supplementary material for: Social threat, neural connectivity, and adolescent mental health: a population-based longitudinal study
Source: Psychol Med. 2025 Sep 18;55:e275. doi: 10.1017/S0033291725101384 (PMC13054912; doi:10.1017/S0033291725101384)
Supplement: Tsomokos et al. supplementary material [file S0033291725101384sup001.docx]

**Supplementary Information (SI) for:**

**Social Threats, Neural Connectivity, and Adolescent Mental Health: A Population-Based Longitudinal Study**

Dimitris I. Tsomokos^1*^, Henning Tiemeier^2^, George M. Slavich^3^, and Divyangana Rakesh^4^

^1^ UCL Institute of Education, Department of Psychology & Human Development, University College London, United Kingdom

^2^ Neuroimaging Department, Institute of Psychology, Psychiatry & Neuroscience, King’s College London, United Kingdom

^3^ Department of Social and Behavioral Sciences, Harvard T.H. Chan School of Public Health, Boston, MA, USA

^4^ Department of Psychiatry and Biobehavioral Sciences, University of California, Los Angeles, CA, USA

***Corresponding Author**: Dr D. I. Tsomokos, UCL Institute of Education, 20 Bedford Way, London WC1H 0AL, UK. Email: [d.tsomokos@ucl.ac.uk](mailto:d.tsomokos@ucl.ac.uk) [ORCID ID 0000-0002-9613-7823]

**This file includes:**

Supporting text

Tables S1 to S7

Figures S1 to S3

SI References

ABCD study functional imaging data

Resting-state functional magnetic resonance imaging (rs-fMRI) data were collected using harmonized protocols across all imaging sites participating in the ABCD Study, as described in detail elsewhere [1]. To ensure sufficient low-motion data for reliable connectivity analyses, participants first completed a few eyes-open, resting-state scans, each lasting around 5 minutes, resulting in at least 8 minutes of higher quality (i.e., low motion) data. Preprocessing of the rs-fMRI data was conducted by the ABCD Data Analysis and Informatics Core using a standardized pipeline that included steps such as motion correction, normalization, and spatial smoothing; fMRI time courses were then projected onto the cortical surface using FreeSurfer to enhance anatomical precision. Functional connectivity both within and between different networks was assessed by calculating correlation coefficients between time courses, following the Gordon parcellation scheme [2], which segments the cortex into networks comprised of functionally similar regions. In this exploratory study, we focused on 5 predefined resting-state networks: the Default Mode Network (DMN), Dorsal Attention Network (DAN), Fronto-Parietal Network (FPN), Cingulo-Opercular Network (CON), Salience Network (SN). These 5 networks yielded 15 connectivity variables, including 10 between-network and 5 within-network variables that capture the average correlation between all pairs of regions within each of these networks.

Sample bias and missingness in the data

At baseline (i.e., when cohort members in ABCD were approximately 10 years of age), there were $11,868$ participants in the survey. The inclusion criteria for the final analytic sample ($N=8,690$), as explained in the main manuscript, were that (a) there were complete records and valid, error-free measurements in the baseline neuroimaging variables; and, (b) that there was participation in the 6-month follow-up wave and complete records were available for the youth-reported total mental health symptom scores in that wave. Two more participants were also excluded from the final sample as they were assigned intersex at birth and the group size was too small to be included in the analysis (statistically underpowered). A flowchart of this sample selection process is provided below (Figure S1).

**Figure S1. Flowchart of the sample selection criteria (for the main analytic sample).**

**
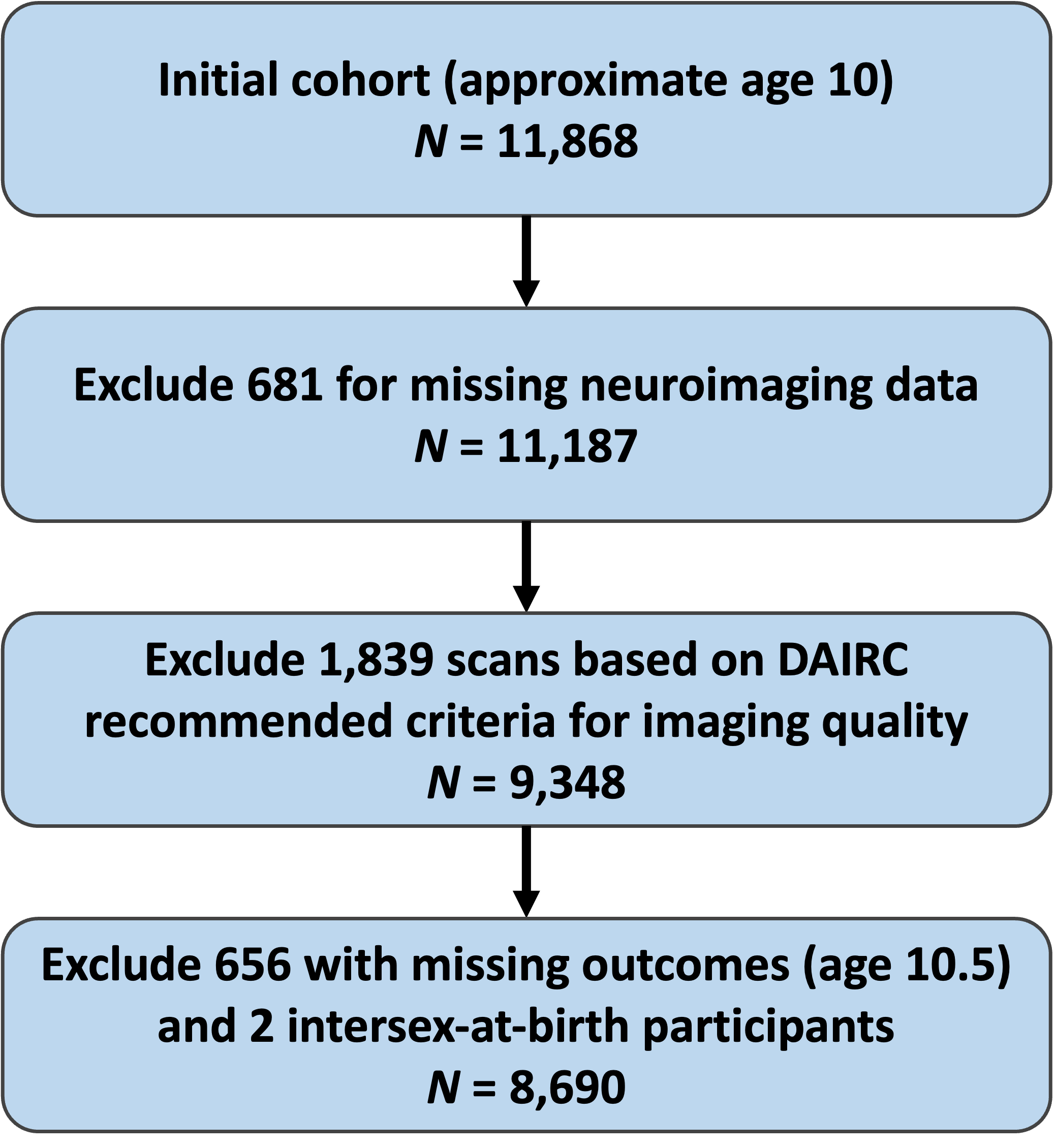
**

Among the $8,690$ participants who remained in the analytic sample (50% female, 45% non-White), there was either none or low missingness in all the variables ($<5\%$ in each variable and $<20\%$ overall), as can be seen in Table S1. The pattern of missing data was not Missing Completely at Random (MCAR) but Missing at Random (MAR) instead, as shown in the Supplemental Online Material [3] via Little’s MCAR test [4] and related analyses. We treat this low amount of MAR missing data using the full information likelihood (FIML) method using all the available data [5, 6]. In line with previous studies on large birth cohort surveys such as the ABCD [7, 8], a sample bias analysis (Table S1) shows that higher socioeconomic status, female, and White cohort members were more likely to participate in follow-up waves and complete mental health data, and therefore be included in the sample (Cohen’s $d=0.14$ for female, and $d=0.27$ for White participants); similarly, the final sample included more parents with a higher educational degree ($d=0.23$) and fewer families living in deprived areas ($d=-0.18$).

Correlations among numerical variables

Table S2 presents the Pearson correlation coefficients between the main numerical variables (confounders and exogenous variables) in the structural regression models. Note that the total social threats variable (as the sum of the three separate environmental exposures of the normalized family conflict, school, and neighborhood) is correlated with each of the three separate exposures in the range between $r=.55$ and $r=.60, p<.001$. The frequency distribution of the primary exposure (total social threats) in our study is shown in Figure S2.

Statistical assumptions

Guided by commonly accepted recommendations [9], we ensured that key assumptions hold for the main structural regression models, starting from the fact that the models were highly powered. In addition, associations between predictors, outcomes, and covariates were linear and there was no collinearity among them; the main exposure (social threats) and outcomes (total mental health, internalizing, externalizing, and attention problems) were numerical, and their residuals were normally distributed. Figure S3 shows most of these assumptions graphically, for the corresponding general linear model (for which the assumptions are, in fact, more restrictive than those needed for the structural equations without latent variables that are used here).

Code in R and Supplemental Online Material (SOM)

The complete output of the statistical analysis and code using R [10] (version 4.4.1 on aarch64-apple-darwin20) has been provided as an html document and published permanently on the Open Science Framework repository as Supplemental Online Material (SOM) [3]. This document includes all the results with higher numerical accuracy (i.e. prior to any rounding in the tables and text), exact *p*-values, before and after controlling for the false discovery rate. It also includes details on the ABCD variables used, including their labels, ranges, descriptive statistics and plots, and complete information on the structural regression models (e.g., standardized coefficients, confidence intervals, model fit indices where relevant, etc.)

**Secondary analyses on the specificity of effects**

1. *Specificity of mental health problems*: In additional models, we broke down the primary outcome variable (total mental health problems) into its constituent subscales—namely, internalizing, externalizing, and attention problems.
2. *Specificity of social threat perceptions*: In additional models, we broke down the primary exposure variable (social threat perceptions) into threats arising from family conflict, the school environment, or the neighborhood environment, and considered them in the same model to assess associations with functional connectivity, thus controlling for each other. For significant associations, we then tested whether functional connectivity mediated the association of the relevant social threat variable and mental health (while controlling for the other types of social threats).

**Secondary analysis with a longer timeframe**

Finally, we shifted the timeframe for all models by two years, in which case we had:

*Time 1 (social threats and rs-fMRI scans, age 10) 🡪 Time 2 (outcomes, age 12.5)*.

Although we refitted the models and rerun all the analyses in this setup [3], the primary purpose of this step was to establish whether any findings on the role of brain connectivity in the link between social threats and later mental health difficulties persisted over longer periods of time, thus informing the timeframe for potential clinical interventions.

Formal comparison of path coefficients in Table 3 (Main)

In the additional analysis on specificity of social threat perceptions, we noted that the regression coefficient for the path from family conflict to later mental health problems was stronger than those for school and neighborhood unsafety. A comparison between them can be made with formal methods [6, 11], and we follow E. Klopp [12] to do this. For instance, in the mediation model involving the DMN (see SOM, Appendix 5a, “Which path is stronger” [3]), we find that the Wald test between family conflict and unsafe school yields $W=66.58, p<0.001$, while between family conflict and unsafe neighborhood it yields $W=104.50, p<0.001$. Finally, the comparison between unsafe school and neighborhood also shows that these two paths are marginally different, with $W=4.21, p=0.040$.

**Table S1.** Demographic characteristics and analytic sample bias: comparison between the analytic sample and the rest of the ABCD survey at baseline (approximate age 10 years).

| **Characteristic** | **Rest of sample at baseline (*N* = 3,178)** | **Analytic sample at baseline (*N* = 8,690)** | ***p*-value** |
| --- | --- | --- | --- |
| Exact age (months), Mean (SD) | 118 (7) | 119 (8) | <0.001 |
| *(Missing)* | 1 | 0 |  |
| Sex, n (%) |  |  | <0.001 |
| *Female* | 1,359 (43) | 4,318 (50) |  |
| *Male* | 1,816 (57) | 4,372 (50) |  |
| *Intersex* | 3 (<0.1) | 0 (0) |  |
| Race/ethnicity, n (%) |  |  | <0.001 |
| *Asian* | 77 (2.4) | 175 (2.0) |  |
| *Black* | 673 (21) | 1,111 (13) |  |
| *Hispanic* | 704 (22) | 1,706 (20) |  |
| *Other* | 347 (11) | 900 (10) |  |
| *White non-Hispanic* | 1,377 (43) | 4,796 (55) |  |
| *(Missing)* | 0 | 2 |  |
| Area deprivation, Mean (SD) | 44 (29) | 39 (26) | <0.001 |
| *(Missing)* | 291 | 588 |  |
| Parental age (years), Mean (SD) | 39 (7) | 40 (7) | <0.001 |
| *(Missing)* | 36 | 55 |  |
| Parental education, n (%) |  |  | <0.001 |
| *1 (Higher education degree)* | 1,431 (45) | 4,892 (56) |  |
| *0 (No degree)* | 1,740 (55) | 3,788 (44) |  |
| *(Missing)* | 7 | 10 |  |
| Parental mental health, Mean (SD) | 22 (19) | 21 (18) | 0.024 |
| *(Missing)* | 2 | 2 |  |
| Unsafe neighborhood, n (%) |  |  | <0.001 |
| *0 (No)* | 2,720 (86) | 7,887 (91) |  |
| *1 (Yes)* | 447 (14) | 790 (9.1) |  |
| *(Missing)* | 11 | 13 |  |
| Unsafe school, n (%) |  |  | <0.001 |
| *0 (No)* | 2,854 (90) | 8,164 (94) |  |
| *1 (Yes)* | 312 (9.9) | 513 (5.9) |  |
| *(Missing)* | 12 | 13 |  |
| Family conflict, Mean (SD) | 2 (2) | 2 (2) | <0.001 |
| *(Missing)* | 12 | 12 |  |
| Social threats (total score), Mean (SD) | 0.48 (0.60) | 0.37 (0.49) | <0.001 |
| *(Missing)* | 14 | 14 |  |
| Imaging: fMRI motion, Mean (SD) | 0.58 (0.56) | 0.23 (0.22) | <0.001 |
| *(Missing)* | 681 | 0 |  |
| Imaging: Scanner name, n(%) |  |  | <0.001 |
| Achieva dStream Philips | 341 (14) | 565 (6.5) |  |
| Discovery MR750 GE | 710 (28) | 2,093 (24) |  |
| Ingenia Philips | 174 (7.0) | 327 (3.8) |  |
| Prisma fit Siemens | 853 (34) | 3,166 (36) |  |
| Prisma Siemens | 419 (17) | 2,539 (29) |  |
| *(Missing)* | 681 | 0 |  |

*Note.* Welch two sample *t*-test and Pearson’s chi-squared test used for the numerical and categorical variables, respectively.

**Table S2.** Correlation matrix for numerical variables in the fully adjusted model.

|  | (1) | (2) | (3) | (4) | (5) |
| --- | --- | --- | --- | --- | --- |
| Parental mental health (1) |  |  |  |  |  |
| Unsafe school (2) | **.08** |  |  |  |  |
| Unsafe neighborhood (3) | **.10** | **.24** |  |  |  |
| Family conflict (4) | **.12** | **.20** | **.17** |  |  |
| Area deprivation (5) | **.16** | **.13** | **.24** | **.11** |  |
| Social threats (6) | **.13** | **.55** | **.59** | **.60** | **.23** |

*Note.* Pearson’s correlation coefficients (values in bold correspond to $p<.05$, two-sided t-test with Bonferroni correction for multiple comparisons)

**Table S3.** Results for the structural equation models testing if the within-network connectivity mediates the association between social threat perceptions at baseline and mental health problems 6 months later ($N=8,690$, adjusted, imputed; full version of Table 2 for the four within-network connectivity variables that remained significant in the analysis of Table 1).

|  | **DMN** | **DAN** | **FPN** | **CON** |
| --- | --- | --- | --- | --- |
|  | Coeff*^1^* (CI)*^2^* | Coeff*^1^* (CI)*^2^* | Coeff*^1^* (CI)*^2^* | Coeff*^1^* (CI)*^2^* |
| **Total Mental Health Problems** |  |  |  |  |
| Social threats  *(c-path)* | **2.90**^***^ (2.62 to 3.18) | **2.90**^***^ (2.63 to 3.18) | **2.90**^***^ (2.62 to 3.18) | **2.90**^***^ (2.63 to 3.18) |
| Sex: Female | -0.74^***^ (-0.95 to -0.53) | -0.80^***^ (-1.01 to -0.59) | -0.77^***^ (-0.98 to -0.56) | -0.79^***^(-1.00 to -0.58) |
| Area deprivation | 0.01^***^ (0.01 to 0.02) | 0.01^***^ (0.01 to 0.02) | 0.01^***^ (0.01 to 0.02) | 0.01^***^ (0.01 to 0.02) |
| Parental education | -0.61^***^ (-0.85 to -0.37) | -0.61^***^ (-0.85 to -0.38) | -0.63^***^ (-0.86 to -0.39) | -0.60^***^ (-0.84 to -0.37) |
| Parental mental health | 0.04^***^ (0.03 to 0.05) | 0.04^***^ (0.03 to 0.04) | 0.04^***^ (0.03 to 0.04) | 0.04^***^ (0.03 to 0.04) |
| DMN *(b-path)* | **-3.95**^***^ (-5.83 to -2.07) |  |  |  |
| DAN *(b-path)* |  | -2.15^**^ (-3.72 to -0.59) |  |  |
| FPN *(b-path)* |  |  | **-3.37**^***^ (-5.22 to -1.51) |  |
| CON *(b-path)* |  |  |  | -1.55^*^ (-3.08 to -0.03) |
| **Functional connectivity** |  |  |  |  |
| Social threats  *(a-path)* | **-0.00**^**^ (-0.01 to -0.00) | -0.00^*^ (-0.01 to -0.00) | **-0.01**^***^(-0.01 to -0.00) | -0.01^***^(-0.01 to -0.00) |
| Sex: Female | 0.02^***^ (0.01 to 0.02) | 0.00 (0.00 to 0.01) | 0.01^***^ (0.01 to 0.01) | 0.01^***^ (0.01 to 0.02) |
| Area deprivation | -0.00^***^ (-0.00 to -0.00) | -0.00^***^ (-0.00 to -0.00) | 0.00 (0.00 to 0.00) | -0.00^***^ (-0.00 to -0.00) |
| Parental education | 0.00 (-0.00 to 0.00) | 0.00 (0.00 to 0.01) | 0.00 (0.00 to 0.00) | 0.01^***^ (0.01 to 0.01) |
| Parental mental health | -0.00 (-0.00 to 0.00) | -0.00 (-0.00 to 0.00) | -0.00 (-0.00 to 0.00) | -0.00 (-0.00 to 0.00) |
| fMRI motion | -0.05^***^ (-0.06 to -0.05) | -0.04^***^ (-0.05 to -0.04) | -0.03^***^ (-0.04 to -0.03) | -0.06^***^ (-0.06 to -0.05) |
| Achieva dStream Philips | -0.01^***^ (-0.02 to -0.01) | 0.00 (-0.01 to 0.00) | 0.01^**^ (0.00 to 0.01) | -0.02^***^ (-0.03 to -0.02) |
| Discovery MR750 GE | -0.03^***^ (-0.04 to -0.03) | -0.01^***^ (-0.02 to -0.01) | -0.02^***^ (-0.02 to -0.02) | -0.03^***^ (-0.04 to -0.03) |
| Ingenia Philips | -0.03^***^ (-0.04 to -0.03) | -0.01 (-0.01 to 0.00) | 0.00 (-0.01 to 0.00) | -0.02^***^ (-0.03 to -0.01) |
| Prisma Siemens | 0.00 (-0.00 to 0.00) | 0.01^**^ (0.00 to 0.01) | -0.01^***^ (-0.01 to -0.00) | 0.01^***^ (0.00 to 0.01) |
|  | **Indirect Effects** Coeff*^1^* (CI)*^2^* | | |  |
| *ab* (indirect) | **0.02**^**^ (0.00 to 0.03) | 0.01 (-0.00 to 0.02) | **0.02**^**^ (0.00 to 0.03) | 0.01 (-0.00 to 0.02) |
|  | **Fit Indices** *^3^* | | |  |
| Robust CFI | 0.99 | 0.99 | 0.99 | 0.99 |
| Robust TLI | 0.97 | 0.95 | 0.95 | 0.96 |
| Robust RMSEA | 0.02 | 0.02 | 0.02 | 0.02 |
| SRMR | 0.01 | 0.01 | 0.01 | 0.01 |
| *^1^* Unstandardized coefficients (^***^p < 0.001; ^**^p < 0.01; ^*^p < 0.05; indirect effects in bold remained significant after controlling for the FDR across all 7 models, i.e. all the models included in Tables S3 and S4). \| *^2^* 95% Confidence intervals. \| *^3^* CFI = comparative fit index; TLI = Tucker–Lewis index; RMSEA = root mean square error of approximation; SRMR = standardized root mean square residual. | | | | |

**Table S4.** Results for the structural equation models testing whether between-network connectivity mediates the association between social threat perceptions at baseline and total mental health problems 6 months later ($N=8,690$, adjusted, imputed; full version of Table 2 for the three significant between-network connectivity variables of Table 1).

|  | **DMN-DAN** | **DMN-CON** | **FPN-CON** |
| --- | --- | --- | --- |
|  | Coeff*^1^* (CI)*^2^* | Coeff*^1^* (CI)*^2^* | Coeff*^1^* (CI)*^2^* |
| **Total Mental Health Problems** |  |  |  |
| Social threats *(c)* | **2.88**^***^ (2.60 to 3.16) | **2.89**^***^ (2.62 to 3.18) | **2.90**^***^ (2.62 to 3.18) |
| Sex: Female | -0.75^***^ (-0.96 to -0.54) | -0.72^***^ (-0.93 to -0.50) | -0.79^***^ (-1.0 to -0.58) |
| Area deprivation | 0.01^***^ (0.01 to 0.02) | 0.01^***^ (0.01 to 0.02) | 0.01^***^ (0.01 to 0.02) |
| Parental education | -0.60^***^ (-0.84 to -0.37) | -0.62^***^ (-0.86 to -0.38) | -0.63^***^ (-0.87 to -0.39) |
| Parental mental health | 0.04^***^ (0.03 to 0.04) | 0.04^***^ (0.03 to 0.04) | 0.04^***^ (0.03 to 0.04) |
| DMN-DAN (*b*) | **5.42**^***^ (3.44 to 7.40) |  |  |
| DMN-CON (*b*) |  | **5.62**^***^ (3.65 to 7.58) |  |
| FPN-CON (*b*) |  |  | **4.04**^**^ (1.75 to 6.32) |
| **Functional connectivity** |  |  |  |
| Social threats *(a)* | **0.01**^***^ (0.00 to 0.01) | **0.00**^**^ (0.00 to 0.01) | **0.00**^**^ (0.00 to 0.01) |
| Sex: Female | -0.01^***^ (-0.01 to -0.01) | -0.02^***^ (-0.02 to -0.01) | -0.00^***^ (-0.01 to -0.00) |
| Area deprivation | 0.00^***^ (0.00 to 0.00) | 0.00 (-0.00 to 0.00) | 0.00^*^ (0.00 to 0.00) |
| Parental education | -0.00 (-0.01 to 0.00) | 0.00 (-0.00 to 0.00) | 0.00^*^ (0.00 to 0.00) |
| Parental mental health | 0.00^*^ (0.00 to 0.00) | 0.00^**^ (0.00 to 0.00) | 0.00 (0.00 to 0.00) |
| fMRI motion | 0.05^***^ (0.05 to 0.06) | 0.06^***^ (0.05 to 0.06) | 0.02^***^ (0.02 to 0.03) |
| Achieva dStream Philips | 0.02^***^ (0.01 to 0.02) | -0.01^***^ (-0.01 to -0.00) | 0.00 (0.00 to 0.01) |
| Discovery MR750 GE | 0.02^***^ (0.02 to 0.02) | 0.00 (0.00 to 0.01) | -0.01^***^ (-0.01 to -0.01) |
| Ingenia Philips | 0.04^***^ (0.03 to 0.05) | 0.02^***^ (0.02 to 0.03) | 0.02^***^ (0.01 to 0.02) |
| Prisma Siemens | -0.01^***^ (-0.01 to -0.01) | -0.01^***^ (-0.01 to -0.00) | -0.00^*^ (-0.01 to -0.00) |
|  | **Indirect Effects** Coeff*^1^* (CI)*^2^* | | |
| *ab* (indirect) | **0.03**^***^ (0.01 to 0.05) | **0.02**^*^ (0.00 to 0.03) | **0.01**^*^ (0.00 to 0.02) |
|  | **Fit Indices** *^3^* | | |
| Robust CFI | 0.99 | 0.99 | 0.98 |
| Robust TLI | 0.97 | 0.97 | 0.94 |
| Robust RMSEA | 0.02 | 0.02 | 0.02 |
| SRMR | 0.01 | 0.00 | 0.01 |
| *^1^* Unstandardized coefficients (^***^p < 0.001; ^**^p < 0.01; ^*^p < 0.05; indirect effects in bold remained significant after controlling for the FDR across all 7 models, i.e. all the models included in Tables S3 and S4). \| *^2^* 95% Confidence intervals. \| *^3^* CFI = comparative fit index; TLI = Tucker–Lewis index; RMSEA = root mean square error of approximation; SRMR = standardized root mean square residual. | | | |

**Table S5.** Results for the structural equation models testing whether within-network connectivity in the focal networks mediates the association between social threats at baseline and attention problems 6 months later ($N=8,690$, adjusted, imputed).

|  | **Within DMN** | **Within DAN** | **Within FPN** | **Within CON** |
| --- | --- | --- | --- | --- |
|  | Coeff*^1^* (CI)*^2^* | Coeff*^1^* (CI)*^2^* | Coeff*^1^* (CI)*^2^* | Coeff*^1^* (CI)*^2^* |
| **Total Mental Health Problems** |  |  |  |  |
| Social threats  *(c-path)* | **1.01**^***^ (0.88 to 1.13) | **1.01**^***^ (0.89 to 1.13) | **1.01**^***^ (0.89 to 1.13) | **1.01**^***^ (0.89 to 1.13) |
| DMN *(b-path)* | **-2.31**^***^ (-3.19 to -1.44) |  |  |  |
| DAN *(b-path)* |  | -1.02^**^ (-1.76 to -0.27) |  |  |
| FPN *(b-path)* |  |  | **-2.09**^***^ (-2.98 to -1.19) |  |
| CON *(b-path)* |  |  |  | **-1.08**^**^ (-1.81 to -0.36) |
| **Functional connectivity** |  |  |  |  |
| Social threats  *(a-path)* | **-0.00**^**^ (-0.01 to -0.00) | -0.00^*^ (-0.01 to -0.00) | **-0.01**^***^(-0.01 to -0.00) | -0.01^***^(-0.01 to -0.00) |
|  | **Indirect and Total Effects** – Standardized Estimates | | |  |
| $\alpha\beta$ (indirect) | **0.002**^**^ | 0.001 | **0.002**^**^ | **0.001**^*^ |
| $\alpha\beta+\gamma$ (total) | 0.002 + 0.198 | 0.001 + 0.200 | 0.002 + 0.199 | 0.001 + 0.199 |
|  | **Fit Indices** *^3^* | | |  |
| Robust CFI | 0.99 | 0.99 | 0.98 | 0.98 |
| Robust TLI | 0.95 | 0.95 | 0.91 | 0.93 |
| Robust RMSEA | 0.03 | 0.02 | 0.03 | 0.03 |
| SRMR | 0.01 | 0.01 | 0.01 | 0.01 |
| *^1^* Unstandardized coefficients (^***^*p* < 0.001; ^**^*p* < 0.01; ^*^*p* < 0.05; indirect effects in bold remained significant after controlling for the FDR across all 7 models, i.e. all the models included in Tables S3 and S4). \| For details on all covariates consult the SOM, Appendix 3 [3] \| *^2^* 95% Confidence intervals. \| *^3^* CFI = comparative fit index; TLI = Tucker–Lewis index; RMSEA = root mean square error of approximation; SRMR = standardized root mean square residual. | | | | |

**Table S6.** Results for the structural equation models testing whether between-network connectivity mediates the association between social threats at baseline and attention problems 6 months later ($N=8,690$, adjusted, imputed).

|  | **DMN-DAN** | **DMN-CON** | **FPN-CON** |
| --- | --- | --- | --- |
|  | Coeff*^1^* (CI)*^2^* | Coeff*^1^* (CI)*^2^* | Coeff*^1^* (CI)*^2^* |
| **Total Mental Health Problems** |  |  |  |
| Social threats *(c)* | **1.00**^***^ (0.88 to 1.12) | **1.00**^***^ (0.88 to 1.12) | **1.01**^***^ (0.89 to 1.13) |
| DMN-DAN (*b*) | **2.77**^***^ (1.83 to 3.71) |  |  |
| DMN-CON (*b*) |  | **3.04**^***^ (2.12 to 3.96) |  |
| FPN-CON (*b*) |  |  | **2.19**^***^ (1.09 to 3.29) |
| **Functional connectivity** |  |  |  |
| Social threats *(a)* | **0.01**^***^ (0.00 to 0.01) | **0.00**^**^ (0.00 to 0.01) | **0.00**^**^ (0.00 to 0.01) |
|  | **Indirect and Total Effects** – Standardized Estimates | | |
| $\alpha\beta$ (indirect) | **0.003**^**^ | **0.002**^*^ | **0.001**^*^ |
| $\alpha\beta+\gamma$ (total) | 0.003 + 0.197 | 0.002 + 0.198 | 0.001 + 0.199 |
|  | **Fit Indices** *^3^* | | |
| Robust CFI | 0.99 | 0.99 | 0.97 |
| Robust TLI | 0.95 | 0.94 | 0.87 |
| Robust RMSEA | 0.03 | 0.02 | 0.03 |
| SRMR | 0.01 | 0.01 | 0.01 |
| *^1^* Unstandardized coefficients (^***^*p* < 0.001; ^**^*p* < 0.01; ^*^*p* < 0.05; indirect effects in bold remained significant after controlling for the FDR across all 7 models, i.e. all the models included in Tables S3 and S4). \| For details on all covariates consult the SOM, Appendix 3 [3] \| *^2^* 95% Confidence intervals. \| *^2^* 95% Confidence intervals. \| *^3^* CFI = comparative fit index; TLI = Tucker–Lewis index; RMSEA = root mean square error of approximation; SRMR = standardized root mean square residual. | | | |

**Table S7.** Results of the sensitivity analysis with additional covariates (parent-reported total child mental health problems T-score at baseline and race/ethnicity) in the structural equation models testing whether within-network and between-network functional connectivity mediates the association between social threats at baseline and total mental health problems 6 months later ($N=8,690$, adjusted, imputed). P-values before and after controlling for the false discovery rate are shown (along with the relevant standardized $\alpha\beta$ indirect effect). Full results for these models (adjusting for all the initial confounders as well as child’s race/ethnicity and total mental health problems T-score at baseline) are included in Section D1 of the SOM [3].

| **Brain networks** | **P-value** | **Adjusted P-value** | **Standardized** $\boldsymbol{\alpha\beta}$ |
| --- | --- | --- | --- |
| Default Mode (DMN) | 0.0373906 | 0.0523468 | 0.001 |
| Dorsal Attention (DAN) | 0.1780095 | 0.1780095 | 0.004 |
| Fronto-Parietal (FPN) | 0.0119529 | 0.041835 | 0.001 |
| Cingulo-Opercular (CON) | 0.1105828 | 0.1290133 | 0.001 |
| DMN-DAN | 0.0031554 | 0.0220876 | 0.002 |
| DMN-CON | 0.0184206 | 0.0429815 | 0.002 |
| FPN-CON | 0.0284575 | 0.0498007 | 0.001 |

**Figure S2. Frequency distribution of the total social threat perceptions variable**

**
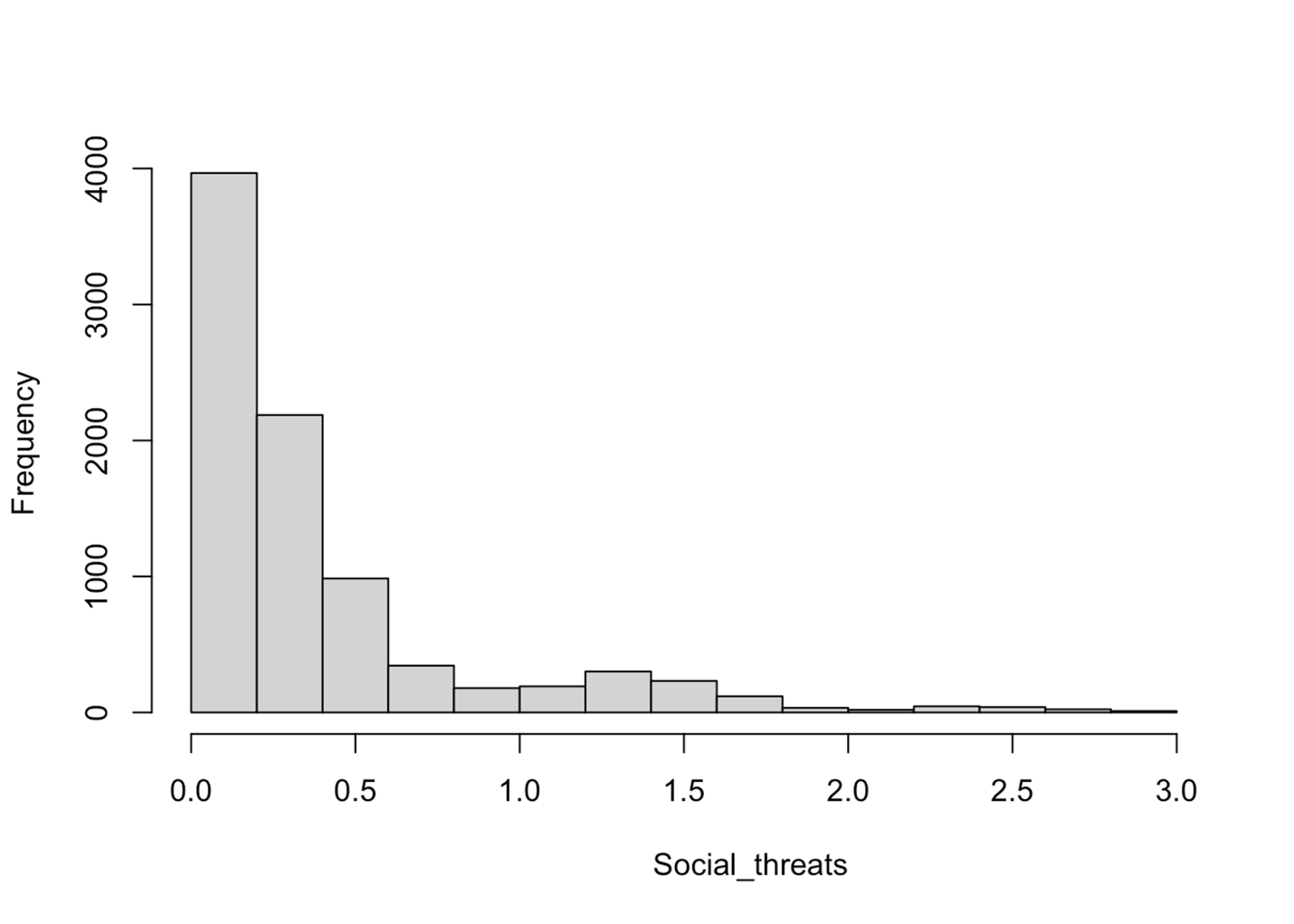
**

**Figure S3. Linearity, collinearity, and normality of residuals in the sample**


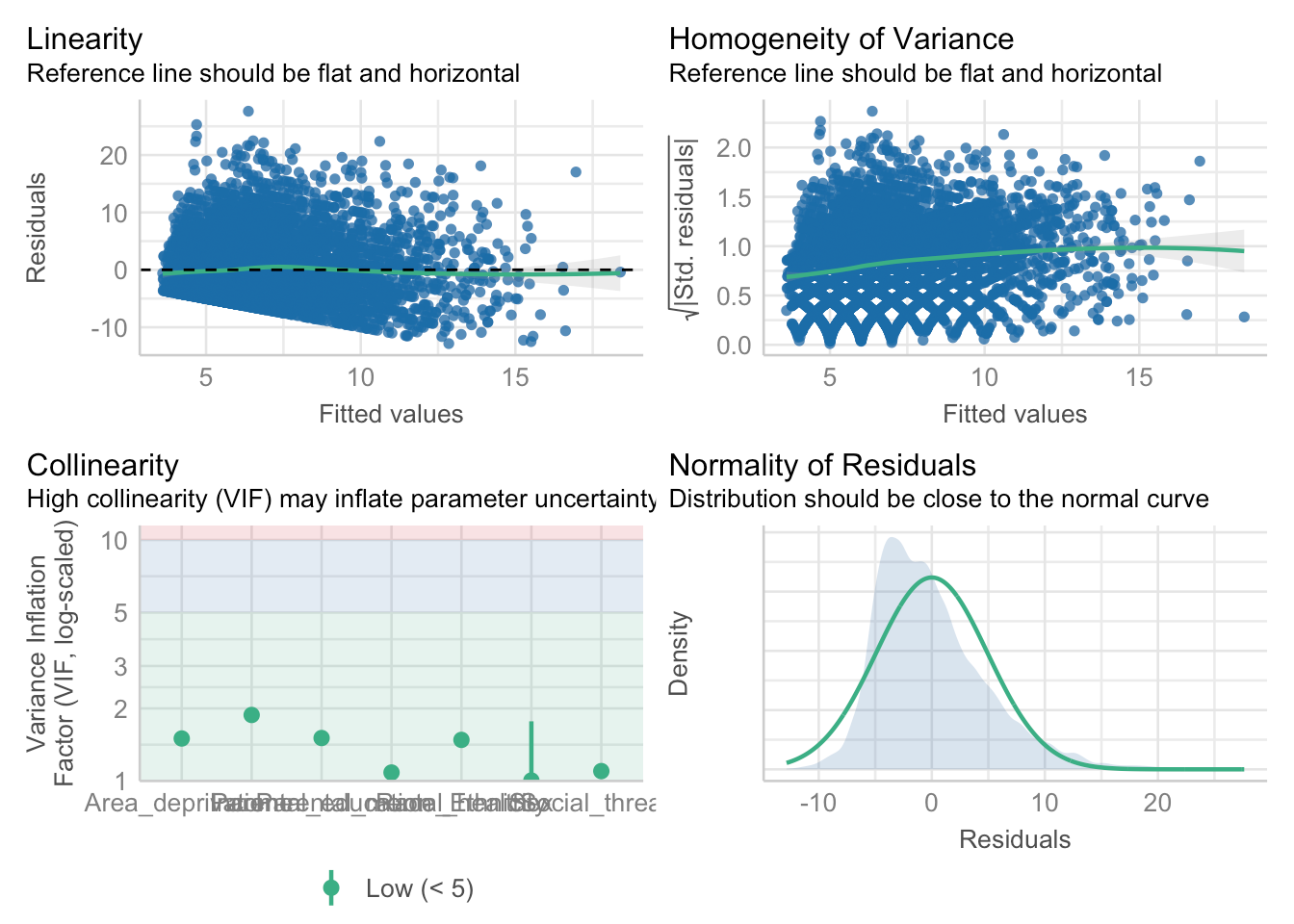


Linearity, homogeneity of variance, collinearity and normality assumption checks for the equivalent general linear model of main interest (Model 1), using the statistical analysis packages detailed in full in the SOM document.

**References**

1. Casey, B.J., et al., *The adolescent brain cognitive development (ABCD) study: imaging acquisition across 21 sites.* Developmental Cognitive Neuroscience, 2018. **32**: p. 43-54.

2. Gordon, E.M., et al., *Generation and evaluation of a cortical area parcellation from resting-state correlations.* Cerebral Cortex, 2016. **26**(1): p. 288-303.

3. SOM. *Supplemental Online Material: Social threats, brain connectivity, and adolescent mental health*. 2025 9/Mar/2025]; Available from: <https://osf.io/7xewv/>.

4. Little, R.J.A., *A Test of Missing Completely at Random for Multivariate Data with Missing Values.* Journal of the American Statistical Association, 1988. **83**(404): p. 1198-1202.

5. Lim, A.J.-M. and M.W.-L. Cheung, *Evaluating FIML and multiple imputation in joint ordinal-continuous measurements models with missing data.* Behavior Research Methods, 2022: p. 1-15.

6. Kline, R.B., *Principles and practice of structural equation modeling*. 2023: Guilford publications.

7. Feldstein Ewing, S.W., et al., *Measuring retention within the adolescent brain cognitive development (ABCD) SM study.* 2022.

8. Saragosa-Harris, N.M., et al., *A practical guide for researchers and reviewers using the ABCD Study and other large longitudinal datasets.* Developmental cognitive neuroscience, 2022. **55**: p. 101115.

9. Streiner, D.L., *Finding our way: an introduction to path analysis.* Can J Psychiatry, 2005. **50**(2): p. 115-22.

10. R.Core.Team, *R: a language and environment for statistical computing*, in *R Foundation for Statistical Computing. URL* [*https://www.R-project.org/*](https://www.R-project.org/). 2021: Vienna, Austria.

11. Rindskopf, D., *Using phantom and imaginary latent variables to parameterize constraints in linear structural models.* Psychometrika, 1984. **49**(1): p. 37-47.

12. Klopp, E., *A tutorial on testing the equality of standardized regression coefficients in structural equation models using Wald tests with lavaan.* The Quantitative Methods for Psychology, 2020. **16**(4): p. 315-333.
